# Prognostic value of radiological T category using conventional MRI in patients with oral tongue cancer: comparison with pathological T category

**DOI:** 10.1007/s00234-024-03345-8

**Published:** 2024-04-12

**Authors:** Masaya Kawaguchi, Hiroki Kato, Tomohiro Kanayama, Hiroyuki Tomita, Akira Hara, Hirofumi Shibata, Takenori Ogawa, Daijiro Hatakeyama, Yoichi Yamada, Tomohiro Ando, Yoshifumi Noda, Fuminori Hyodo, Masayuki Matsuo

**Affiliations:** 1 Department of Radiology, Gifu University, 1-1 Yanagido, Gifu, 501-1194 Japan; 2 Department of Tumor Pathology, Gifu University, Gifu, Japan; 3 Department of Otolaryngology, Gifu University, Gifu, Japan; 4 Department of Oral Maxillofacial Surgery, Gifu University, Gifu, Japan; 5 Center for One Medicine Innovative Translational Research (COMIT), Institute for Advanced Study, Gifu University, Gifu, Japan

**Keywords:** Oral tongue squamous cell carcinoma, Prognosis, Tumor staging, Peritumoral enhancement, MRI

## Abstract

**Purpose:**

This study aimed to compare the radiological tumor (T)-category using multiparametric MRI with the pathological T category in patients with oral tongue squamous cell carcinoma (OTSCC) and to examine which is a better predictor of prognosis.

**Methods:**

This retrospective study included 110 consecutive patients with surgically resected primary OTSCC who underwent preoperative contrast-enhanced MRI. T categories determined by maximum diameter and depth of invasion were retrospectively assessed based on the pathological specimen and multiparametric MRI. The MRI assessment included the axial and coronal T1-weighted image (T1WI), axial T2-weighted image (T2WI), coronal fat-suppressed T2WI, and axial and coronal fat-suppressed contrast-enhanced T1WI (CET1WI). Axial and coronal CET1WI measurements were divided into two groups: measurements excluding peritumoral enhancement (MEP) and measurements including peritumoral enhancement. The prognostic values for recurrence and disease-specific survival after radiological and pathological T categorization of cases into T1/T2 and T3/T4 groups were compared.

**Results:**

The T category of MEP on coronal CET1WI was the most relevant prognostic factor for recurrence [hazard ratio (HR) = 3.30, *p* = 0.001] and the HR was higher than the HR for pathological assessment (HR = 2.26, *p* = 0.026). The T category determined by MEP on coronal CET1WI was also the most relevant prognostic factor for disease-specific survival (HR = 3.12, *p* = 0.03), and the HR was higher than the HR for pathological assessment (HR = 2.02, *p* = 0.20).

**Conclusion:**

The T category determined by MEP on the coronal CET1WI was the best prognostic factor among all radiological and pathological T category measurements.

## Introduction

Oral tongue squamous cell carcinoma (OTSCC) is the eighth most common cancer worldwide [1, 2]. OTSCC accounts for more than 90% of all oral malignancies and is more prevalent in males than females [1, 3]. Even in early-stage disease, tumors recur in 10–25% of cases. In advanced staged disease, the rate of tumor recurrence is approximately 40–60% of cases [4]. According to the eighth edition of the American Joint Committee on Cancer (AJCC) staging manual, T categorization of OTSCC is defined by the maximum diameter and depth of invasion (DOI). A DOI of OTSCC greater than 5 mm and 10 mm is recommended as the gold standard threshold for T1–T3 staging and preventive dissection as follows: T1, tumor ≤ 2 cm with DOI ≤ 5 mm; T2, tumor ≤ 2 cm with DOI > 5 mm and ≤ 10 mm or tumor > 2 cm but ≤ 4 cm with DOI ≤ 10 mm; T3, tumor > 4 cm or any tumor size with DOI > 10 mm. Follow-up after treatment should be considered in cases of OTSCC with DOIs exceeding 5 mm [2, 5–7]. The precise and reproducible measurement of pathological DOI is essential because as lack of adherence to a uniform definition can significantly affect accurate staging; however, the measurement of pathological DOI is sometimes difficult for various reasons [7].

In recent years, the relationship between pathological and radiological DOI has been investigated in many studies and several meta-analyses. Radiological DOI is generally 2–3 mm larger than pathological DOI, resulting in overestimating the clinical T staging [2, 5, 8–18]. The overestimation of radiological DOI is caused by severe local inflammation, peritumoral edema, and local tissue swelling after having a biopsy [9, 10, 16]. In these studies, the gold standard for T staging of OTSCC is based on pathological assessment, and radiological DOI or radiology-based T staging correlates with the pathological referenced standard. However, up to 30% shrinkage of soft tissue may occur after formalin fixation; therefore, the pathological T category is derived from the actual measurement of unfixed tumor in the resected specimen [19]. We thought that radiology-based T staging might be more useful in predicting prognosis than pathological T staging. In addition, the appropriate MRI sequence and imaging plane for the measurement of the maximum radiological diameter and DOI have not been established. To the best of our knowledge, no study has compared pathological T staging with radiological T staging for the ability to predict OTSCC for predicting tumor recurrence and patient survival. Thus, this study aimed to compare the prognostic value of radiological T categorization using multiparametric MRI with pathological T categorization in patients with OTSCC.

## Methods

### Patients

The present study was approved by the human research committee of the institutional review board of our hospital and complies with the guidelines of the Health Insurance Portability and Accountability Act of 1996 and the Declaration of Helsinki. the requirement for informed consent was waived due to the retrospective nature of the study. Consecutive patients with histologically proven OTSCC who underwent preoperative contrast-enhanced MRI between January 2007 and August 2022 were identified using the electronic medical record system at our hospital. The following inclusion criteria were applied: (1) gross total resection, (2) histologically diagnosed cases, and (3) preoperative MRI, including axial T1-weighted image (T1WI), axial T2-weighted image (T2WI), axial fat-suppressed contrast-enhanced T1WI (CET1WI), coronal T1WI, coronal fat-suppressed T2WI, and coronal CET1WI. The exclusion criteria were as follows: (1) prior history of head and neck squamous cell carcinoma, (2) invasion of adjacent structures (cortical bone, skin, masticator space, pterygoid plates, skull base, or internal carotid artery), and (3) inappropriate image quality for evaluation.

### Pathological assessment

A pathologist with 7 years of post-training experience in the pathological diagnosis of OSTCC reviewed all histological specimens based on the eighth edition of the AJCC staging manual and determined the T category based on the maximum diameter and DOI. DOI was defined as measurement from the level of the basement membrane of the closet adjacent normal mucosa to the deepest point of invasion [7, 20].

### MRI imaging

All patients underwent unenhanced and enhanced MRI using a 1.5-T-MRI system (Intera Achieva 1.5 T Pulsar; Philips Medical Systems, Best, The Netherlands) or a 3.0 T-MRI system (Intera Achieva 3.0 T Quasar Dual; Philips Medical Systems, Best, The Netherlands). All MR images were obtained at a section thickness of 3–4 mm with 1-mm intersection gap and a 20 × 20–26 × 26-cm field of view. Axial and coronal T1WIs (TR/TE, 609–827/9–18 ms), axial T2WIs (TR/TE, 3398–5709/90 ms), and coronal fat-suppressed T2WIs (TR/TE, 3330–6670/60–90 ms) were obtained. Axial and coronal fat-suppressed CET1WIs (TR/TE, 630–756/9–18 ms) were obtained after the intravenous injection of 0.1 mmol/kg of gadopentetate dimeglumine (Magnevist, Bayer HealthCare, Leverkusen, Germany) or gadobutrol (Gadavist, Bayer HealthCare, Leverkusen, Germany).

### Imaging assessment

Two radiologists with 23 and 9 years of post-training experience in head and neck imaging reviewed all the images individually. The radiologists were unaware of the clinical information and pathological diagnoses.

The reviewers measured the maximum diameter and DOI on the axial T1WI, axial T2WI, axial CET1WI, coronal T1WI, coronal fat-suppressed T2WI, and coronal CE-T1WI. The maximum diameter and DOI were measured from axial and coronal images and the average value measured by the two radiologists was used.

**Fig. 1:**
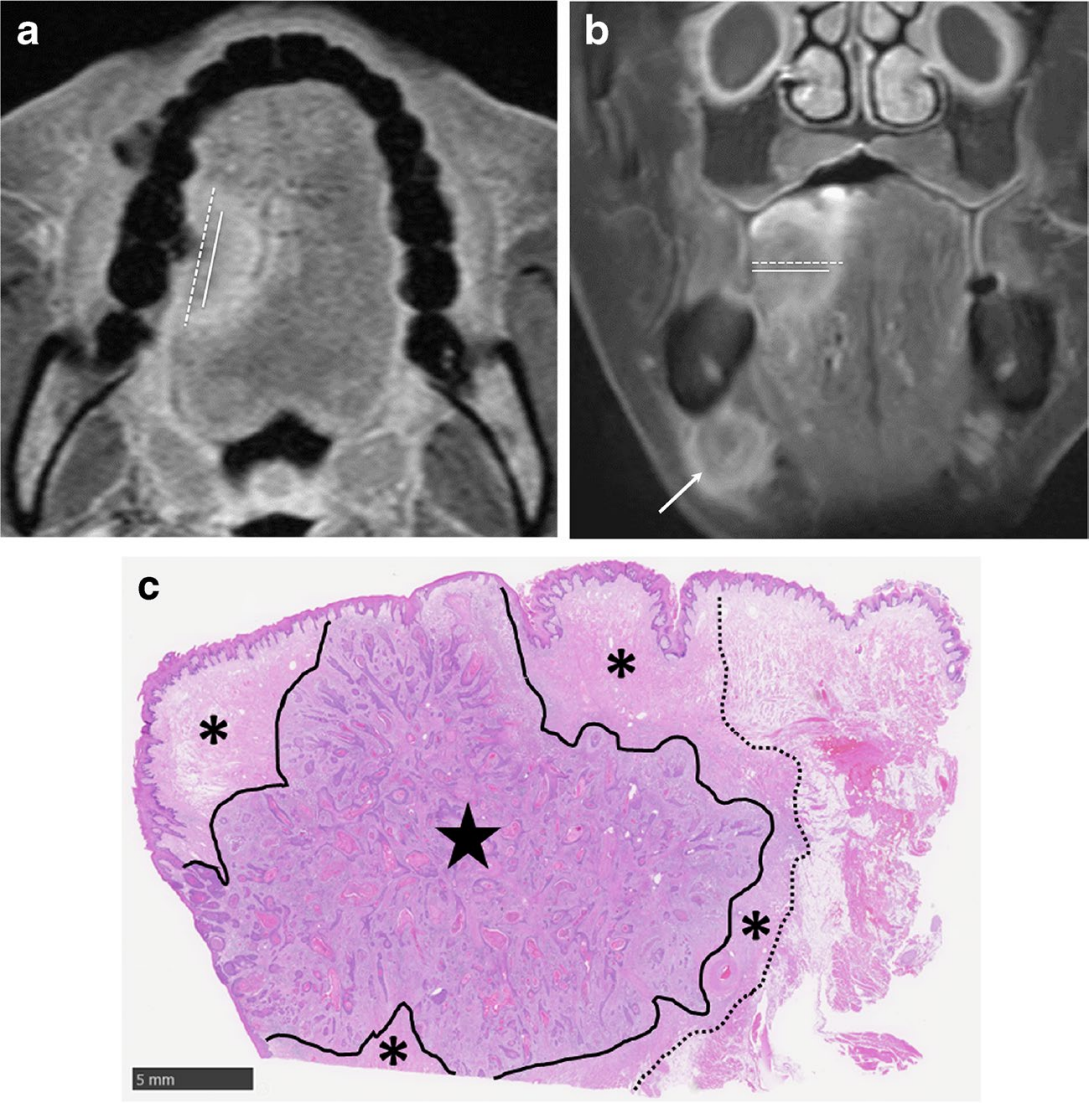
**a** Axial fat-suppressed contrast-enhanced T1-weighted image shows the measurement of the maximum diameter determined by MEP (solid line) and MIP (dotted line). **b** Coronal fat-suppressed contrast-enhanced T1-weighted image shows the measurement of the depth of invasion determined by MEP (solid line) and MIP (dotted line). Right submandibular nodal metastasis is shown (arrow). **c** Histological specimen (H&E stain; scale bar, 5 mm) shows tongue cancer in the central areas (star) and inflammation, vascular enlargement, or edema in the peripheral areas (asterisk)

The DOI was measured at the deepest infiltration point on each image. The DOI was measured from the level of the mucosal surface adjacent to the tumor to the deepest point of tumor invasion [9]. The maximum diameter and DOI on the axial and coronal CET1WI were divided into two groups: measurements including peritumoral enhancement (MIP) and measurements excluding peritumoral enhancement (MEP) (Fig. 1). Peritumoral enhancement was defined as a peripheral stronger enhanced area compared with a less enhanced central area on CET1WI. The less enhanced central area on CET1WI is generally consistent with tumor size on the other sequences. If peritumoral enhancement was not observed, MIP was recorded as the same value as MEP. The maximum diameter and DOI on T1WI and CET1WI were assessed using both the axial and coronal planes; therefore, the greater value was used as the maximum diameter and DOI on the combined axial and coronal images. Finally, T categories were determined for each imaging sequence according to the eighth edition of the AJCC staging manual.

### Statistical analysis

All statistical analyses were performed using SPSS version 24.0 (IBM Corp., Armonk, NY, USA). The correlations of radiological and pathological measurements were assessed by Pearson’s correlation coefficient. Interobserver variability of qualitative assessments between two radiologists was assessed using kappa statistics. The frequency of histologically proven cervical nodal metastasis was compared by Fisher’s exact test between T1/2 and T3/4 cases. The odds ratio and 95% confidence intervals (CIs) of predictive factors for diagnosing lymph node metastasis were calculated by logistic regression models. Log-rank test and Cox proportional hazard regression were conducted for univariate and multivariate analysis to evaluate prognostic factors for recurrence and disease-specific survival between T1/2 and T3/4 cases. *p* values < 0.05 were considered significant.

## Results

We found 130 patients with OTSCC after pathological examination and 20 patients excluded (16 patients with inadequate MRI image and 5 with prior history of head and neck squamous cell carcinoma). This study included 110 patients with OTSCC (71 men; age range, 23–90 years; mean age, 62 years). Thirty-two, 51, 22, and 5 patients were classified into T1, T2, T3, and T4 OTSCC T categories, respectively. Thirty-six patients (33%) had histologically proven cervical nodal metastases and no patient had distant metastasis on initial treatment. All patients had grossly complete removal of the primary tumor and lymph node metastasis at initial surgery. Thirty-nine (35%), 26 (24%), and 59 (54%) patients received chemotherapy, radiation therapy, and radical neck dissection, respectively. Thirty-two patients (29%) experienced recurrence after surgery and 16 (15%) patients died of OTSCC. The median follow-up period was 1315 days (interquartile range [IQR]: 562–2553 days).

The maximum diameters and DOIs are summarized in Table 1.

**Table 1:** The measurements of MD and DOI and Pearson’s coefficient of pathological-radiological maximum diameter and DOI

		MD (mm)	Pearson’s coefficient (*p*)	Kappa value (95% CI)
Pathology		16.6 [22.0–33.0]		
Axial	T1WI	17.0 [10.5–23.9]	0.27 (< 0.01*)	0.75 (0.69–0.81)
	T2WI	16.0 [9.5–22.9]	0.30 (< 0.01*)	0.67 (0.59–0.75)
	CET1WI MEP	16.6 [11.4–23.0]	0.31 (< 0.01*)	0.66 (0.59–0.74)
	CET1WI MIP	18.6 [12.9–26.6]	0.31 (< 0.01*)	0.71 (0.65–0.78)
Coronal	T1WI	14.8 [8.9–20.5]	0.30 (< 0.01*)	0.70 (0.63–0.77)
	FST2WI	15.2 [11.1–20.5]	0.37 (< 0.01*)	0.66 (0.59–0.74)
	CET1WI MEP	14.2 [10.0–18.0]	0.31 (< 0.01*)	0.67 (0.59–0.75)
	CET1WI MIP	16.1 [12.5–23.4]	0.36 (< 0.01*)	0.71 (0.64–0.78)
Combined Axial/Coronal	T1WI	17.8 [12.0–24.8]	0.19 (0.047*)	
	CET1WI MEP	17.0 [12.3–23.6]	0.23 (0.016*)	
	CET1WI MIP	19.4 [14.0–28.2]	0.25 (< 0.01*)	
		DOI (mm)		
Pathology		6.5 [3.1–10.8]		
Axial	T1WI	9.9 [6.2–13.5]	0.79 (< 0.01*)	0.75 (0.69–0.82)
	T2WI	9.5 [5.8–13.1]	0.81 (< 0.01*)	0.68 (0.62–0.76)
	CET1WI MEP	8.9 [6.1–12.7]	0.81 (< 0.01*)	0.71 (0.65–0.78)
	CET1WI MIP	10.6 [7.2–14.8]	0.82 (< 0.01*)	0.73 (0.66–0.80)
Coronal	T1WI	10.2 [6.0–13.6]	0.82 (< 0.01*)	0.73 (0.67–0.79)
	FST2WI	10.0 [6.6–13.8]	0.83 (< 0.01*)	0.71 (0.64–0.77)
	CET1WI MEP	9.1 [6.1–12.9]	0.85 (< 0.01*)	0.71 (0.65–0.77)
	CET1WI MIP	10.9 [7.2–14.8]	0.85 (< 0.01*)	0.75 (0.69–0.81)
Combined Axial/Coronal	T1WI	10.7 [6.6–14.6]	0.81 (< 0.01*)	
	CET1WI MEP	9.8 [6.5–13.3]	0.80 (< 0.01*)	
	CET1WI MIP	11.0 [7.5–15.3]	0.82 (< 0.01*)	

*Note*. *MD*, maximum diameter; *DOI*, depth of invasion; *T1WI*, T1-weighted images; *T2WI*, T2-weighted images; *CET1WI*, fat-suppressed contrast-enhanced T1WI; *FST2WI*, fat-suppressed T2WI; *MEP*, measurement excluding peritumoral enhancement; *MIP*, measurement including peritumoral enhancement

Quantitative data are expressed as medians with interquartile in parentheses

Pearson’s coefficient shows the correlation between pathological and radiological measurements

Kappa value shows interobserver agreement between two radiologists

*p < 0.05, significant difference

The median pathological maximum diameter and DOI were 16.6 mm (IQR: 22.0–33.0) and 6.5 mm (IQR: 3.1–10.8), respectively. The median radiological maximum diameters on the axial, coronal, and combined axial/coronal images were 16.0–18.6 mm, 14.2–16.1 mm, and 17.0–19.4 mm, respectively. The radiological DOIs on the axial, coronal, and axial/coronal images were 9.5–10.6 mm, 9.1–10.9 mm, and 9.8–11.0 mm, respectively. Pearson’s coefficients between the pathological and radiological maximum diameters and DOIs were 0.19–0.37 and 0.79–0.85, respectively. The Kappa value for interobserver agreement between the two radiologists ranged from 0.59 to 0.75.

The relationship between histologically proven cervical nodal metastasis and the T category is summarized in Table 2. Among all radiological and pathological assessments, the frequency of cervical nodal metastasis was significantly higher in T3/T4 compared with the frequency in T1/T2 (*p* < 0.01). The odds ratio for pathological T category was 4.58 (95%CI; 1.83–11.5), and the odds ratios for the radiological T category ranged from 5.36 to 13.0.

**Table 2:** The relationship between histologically proven cervical nodal metastasis and T category

		T1/T2	T3/T4	Odds ratio (95% CI)	*p* value
		N+	N+		
Pathology		20/83 (24)	16/27 (59)	4.58 (1.83–11.5)	< 0.01*
Axial	T1WI	10/69 (14)	26/41 (63)	10.2 (4.06–25.8)	< 0.01*
	T2WI	13/75 (17)	23/35 (66)	9.14 (3.65–22.9)	< 0.01*
	CET1WI-MEP	13/77 (17)	23/33 (70)	11.3 (4.37–29.3)	< 0.01*
	CET1WI-MIP	9/66 (14)	27/44 (61)	10.1 (3.97–25.5)	< 0.01*
Coronal	T1WI	16/83 (19)	20/27 (74)	12.0 (4.32–33.1)	< 0.01*
	FST2WI	16/80 (20)	20/30 (67)	8.00 (3.14–20.4)	< 0.01*
	CET1WI-MEP	22/91 (24)	14/19 (74)	8.78 (2.84–27.1)	< 0.01*
	CET1WI-MIP	16/76 (21)	20/34 (59)	5.36 (2.23–12.9)	< 0.01*
Combined Axial/Coronal	T1WI	9/67 (13)	27/43 (63)	10.9 (4.27–27.7)	< 0.01*
	CET1WI-MEP	11/74 (15)	25/36 (69)	13.0 (5.01–33.8)	< 0.01*
	CET1WI-MIP	8/61 (13)	28/49 (57)	8.83 (3.47–22.5)	< 0.01*

*Note*. *T1WI*, T1-weighted images; *T2WI*, T2-weighted images; *CET1WI*, fat-suppressed contrast-enhanced T1WI; *FST2WI*, fat-suppressed T2WI; *MEP*, measurement excluding peritumoral enhancement; *MIP*, measurement including peritumoral enhancement

Qualitative variables are expressed as raw numbers; numbers in parentheses are proportions followed by percentages

*p < 0.05, significant difference

**Fig. 2:**
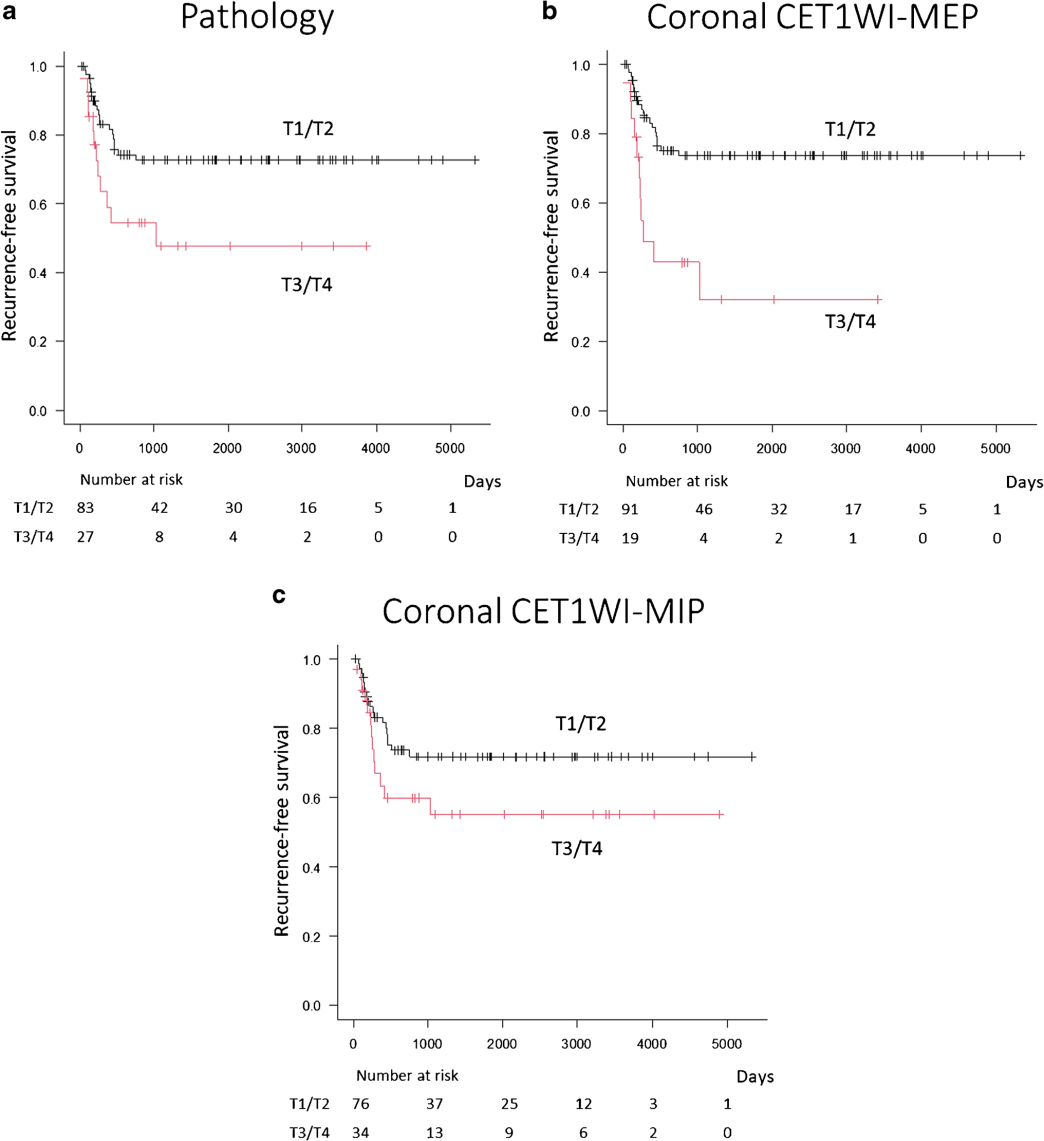
Prognostic impact in patients with OTSCC. Kaplan–Meier survival analysis for recurrence-free survival (T1/T2 vs. T3/T4). The T categories are measured by **a** pathology,** b** coronal CET1WI-MEP, and **c** coronal CET1WI-MIP

The univariable analysis of prognostic factors for recurrence is summarized in Table 3. Significant prognostic factors for recurrence included pathologic T category [hazard ratio (HR); 2.23, *p* = 0.03], axial CET1WI-MEP (2.10, *p* = 0.04), coronal T1WI (2.84, *p* < 0.01), coronal FST2WI (2.26,* p* = 0.02), coronal CET1WI-MEP (3.30, *p* < 0.01), and combined axial/coronal T1WI (2.14, *p* = 0.03) (Fig. 2). However, multivariate analysis revealed no significant prognostic factor for recurrence.

**Table 3:** Univariable analysis of prognostic factors for recurrence

		Recurrence		Hazard ratio (95% CI)	*p* value
		T1/T2	T3/T4		
Pathology		20/83 (24)	12/27 (44)	2.23 (1.10–4.63)	0.03*
Axial	T1WI	17/69 (25)	15/41 (37)	1.80 (0.90–3.60)	0.10
	T2WI	19/75 (25)	13/35 (37)	1.66 (0.82–3.36)	0.16
	CET1WI-MEP	18/77 (23)	14/33 (42)	2.10 (1.05–4.23)	0.04*
	CET1WI-MIP	16/66 (24)	16/44 (36)	1.74 (0.87–3.49)	0.12
Coronal	T1WI	18/83 (22)	14/27 (52)	2.84 (1.41–5.73)	< 0.01*
	FST2WI	19/80 (24)	13/30 (43)	2.26 (1.11–4.58)	0.022*
	CET1WI-MEP	21/91 (23)	11/19 (58)	3.30 (1.59–6.89)	< 0.01*
	CET1WI-MIP	19/76 (25)	13/34 (38)	1.73 (0.86–3.51)	0.12
Combined Axial/Coronal	T1WI	17/67 (25)	15/43 (35)	1.61 (0.80–3.22)	0.18
	CET1WI-MEP	17/74 (23)	15/36 (42)	2.14 (1.07–4.29)	0.03*
	CET1WI-MIP	15/61 (25)	17/49 (35)	1.62 (0.81–3.25)	0.17

*Note*. *T1WI*, T1-weighted images; *T2WI*, T2-weighted images; *CET1WI*, fat-suppressed contrast-enhanced T1WI; *FST2WI*, fat-suppressed T2WI; *MEP*, measurement excluding peritumoral enhancement; *MIP*, measurement including peritumoral enhancement

Qualitative variables are expressed as raw numbers; numbers in parentheses are proportions followed by percentages

*p < 0.05, significant difference

**Fig. 3:**
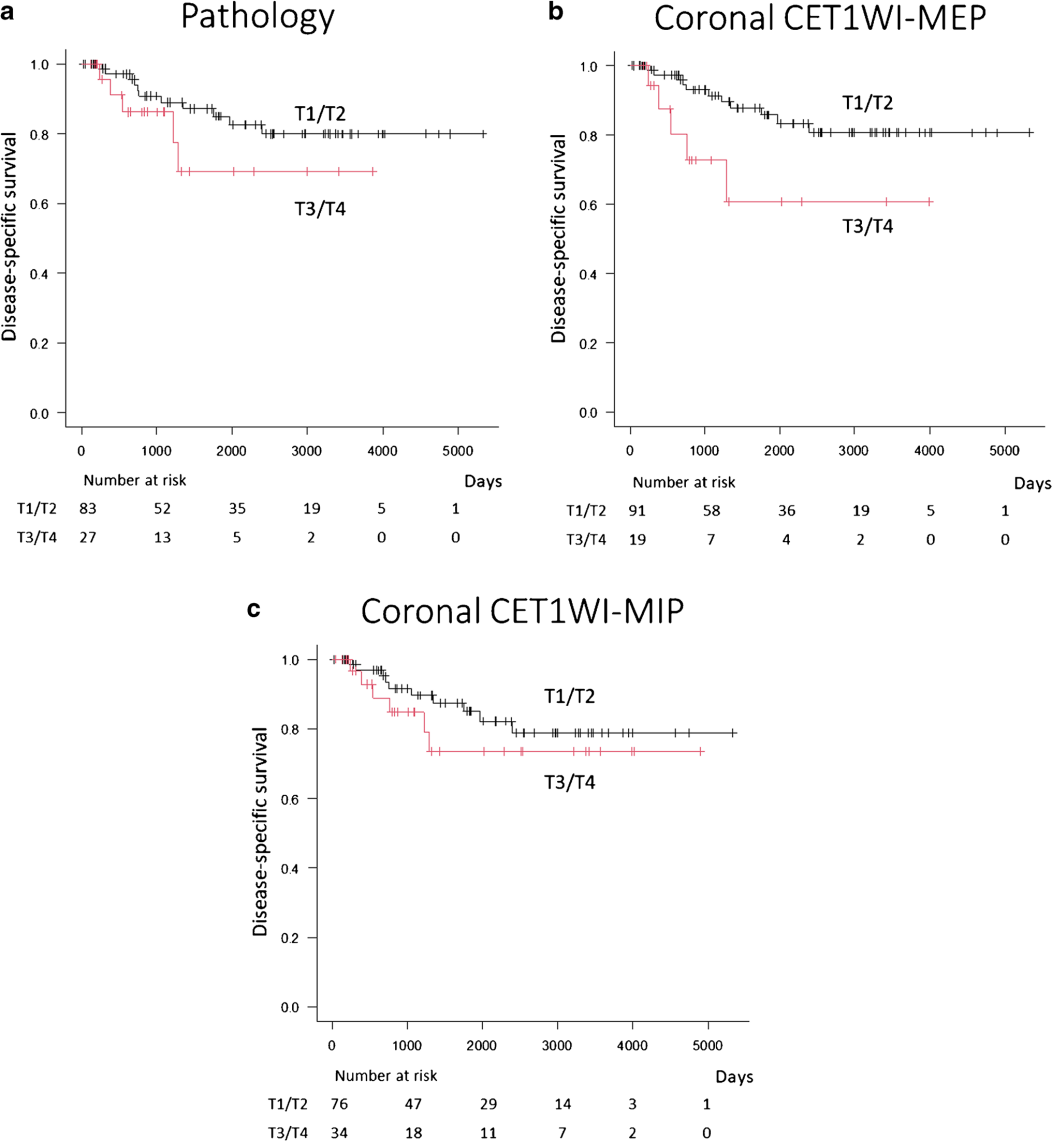
Prognostic impact in patients with OTSCC. Kaplan–Meier survival analysis for disease-specific survival (T1/T2 vs. T3/T4). The T categories are measured by **a** pathology, **b** coronal CET1WI-MEP, and **c** coronal CET1WI-MIP

The univariable analysis of prognostic factors for disease-specific survival and overall survival is summarized in Table 4 and 5. The only significant prognostic factor for disease-specific survival was the T category on coronal CET1WI-MEP (HR; 3.20, *p* = 0.03). The pathological T category was not a significant prognostic factor for disease-specific survival (HR; 2.02, *p* = 0.20) (Fig. 3). However, multivariate analysis revealed no significant prognostic factor for disease-specific survival. No significant prognostic factor for overall survival was observed.

**Table 4:** Univariable analysis of prognostic factors for disease-specific survival

		Mortality		Hazard ratio (95% CI)	*p* value
		T1/T2	T3/T4		
Pathology		11/83 (13)	5/27 (19)	2.02 (0.69–5.90)	0.20
Axial	T1WI	9/69 (13)	7/41 (17)	1.78 (0.66–4.79)	0.26
	T2WI	10/75 (13)	6/35 (17)	1.45 (0.53–4.00)	0.47
	CET1WI- MEP	9/77 (12)	7/33 (20)	2.05 (0.76–5.52)	0.15
	CET1WI-MIP	8/66 (12)	8/44 (18)	1.75 (0.65–4.67)	0.26
Coronal	T1WI	11/83 (13)	5/27 (19)	2.04 (0.74–5.63)	0.17
	FST2WI	10/80 (13)	6/30 (20)	2.07 (0.75–5.69)	0.16
	CET1WI-MEP	11/91 (12)	5/19 (26)	3.20 (1.10–9.32)	0.03*
	CET1WI-MIP	10/76 (13)	6/34 (18)	1.58 (0.57–4.36)	0.38
Combined Axial/Coronal	T1WI	9/67 (13)	7/43 (16)	1.53 (0.57–4.12)	0.40
	CET1WI-MEP	9/74 (12)	7/36 (19)	1.94 (0.72–5.23)	0.19
	CET1WI-MIP	8/61 (13)	8/49 (16)	1.47 (0.55–3.93)	0.44

*Note. T1WI*, T1-weighted images; *T2WI*, T2-weighted images; *CET1WI*, fat-suppressed contrast-enhanced T1WI; *FST2WI*, fat-suppressed T2WI; *MEP*, measurement excluding peritumoral enhancement; *MIP*, measurement including peritumoral enhancement

Qualitative variables are expressed as raw numbers; numbers in parentheses are proportions followed by percentages

*p < 0.05, significant difference

**Table 5:** Univariable analysis of prognostic factors for overall survival

		Mortality		Hazard ratio (95% CI)	*p* value
		T1/T2	T3/T4		
Pathology		14/83 (13)	5/27 (19)	1.64 (0.58–4.63)	0.35
Axial	T1WI	11/69 (13)	8/41 (17)	1.68 (0.67–4.20)	0.27
	T2WI	12/75 (13)	7/35 (17)	1.40 (0.55–3.57)	0.48
	CET1WI- MEP	11/77 (12)	8/33 (20)	1.90 (0.76–4.73)	0.17
	CET1WI-MIP	10/66 (12)	9/44 (18)	1.57 (0.64–3.87)	0.33
Coronal	T1WI	13/83 (13)	6/27 (19)	1.57 (0.59–4.12)	0.36
	FST2WI	12/80 (13)	7/30 (20)	2.02 (0.79–5.14)	0.14
	CET1WI-MEP	14/91 (12)	5/19 (26)	2.54 (0.90–7.14)	0.067
	CET1WI-MIP	11/76 (13)	8/34 (18)	1.87 (0.75–4.66)	0.18
Combined Axial/Coronal	T1WI	11/67 (13)	8/43 (16)	1.43 (0.58–3.57)	0.44
	CET1WI-MEP	11/74 (12)	8/36 (19)	1.80 (0.72–4.49)	0.21
	CET1WI-MIP	9/61 (13)	10/49 (16)	1.60 (0.65–3.93)	0.31

*Note*. *T1WI*, T1-weighted images; *T2WI*, T2-weighted images; *CET1WI*, fat-suppressed contrast-enhanced T1WI; *FST2WI*, fat-suppressed T2WI; *MEP*, measurement excluding peritumoral enhancement; *MIP*, measurement including peritumoral enhancement

Qualitative variables are expressed as raw numbers; numbers in parentheses are proportions followed by percentages

## Discussion

The T category determined based on the coronal CET1WI-MEP was the most relevant prognostic factor for tumor recurrence (HR, 3.30), and the HR for coronal CET1WI-MEP was higher than the HR based on the pathological assessment (HR, 2.26). The T category of coronal CET1WI-MEP was also the most relevant prognostic factor for disease-specific survival (HR, 3.12), and the HR for coronal CET1WI-MEP was higher than the HR for pathological assessment (HR, 2.02).

The radiological DOI on CET1WI-MEP and CET1WI-MIP correlated the best with the pathological assessment. In previous studies, the correlations between radiological and pathological DOI varied considerably. Tang et al. reported that CET1WI was slightly more accurate than T2WI [2]. Baba et al. reported that T1WI had the most accurate correlation, followed by CET1WI and T2WI [8]. Takamura et al. reported that axial CET1WI was more accurate than axial T2WI, and coronal T2WI was more accurate than coronal CET1WI [5]. These variations may stem from the different imaging planes used for the evaluations, only the axial plane, only the coronal plane, and the combined axial and coronal planes.

In the present study, the radiological DOI measured from coronal images tended to correlate better with the pathological DOI than the DOI from axial or combined axial/coronal images. Although many studies evaluated the radiological DOI using both axial and coronal images [8, 9, 21], few studies compared radiological DOI between axial and coronal images [5]. No consensus concerning the optimal imaging plane for evaluating radiological DOI and maximum diameter has been reached. Takamura et al. reported that the mean DOI measured on the coronal CET1WI and T2WI correlated better with the pathological DOI than the DOI measured on the axial CET1WI and T2WI (0.79–0.83 vs. 0.66–0.73) [5]. This difference may arise because the specimen is excised in the cross-section closest to the coronal section [5]. Our results are similar to those of the previous study because the pathological specimen in this study was also excised in the coronal section. Therefore, the radiological maximum diameter and DOI of coronal images should correlate best with the pathological maximum diameter and DOI.

Although the correlation coefficients for CET1WI-MEP and CET1WI-MIP with the pathological-radiological measurements were similar, CET1WI-MEP was superior to CET1WI-MIP for predicting recurrence and disease-specific survival. Histopathologically, peritumoral enhancement may be caused by edematous changes and reactive inflammation in the surrounding tissue [5, 22]. Many studies reported the assessment of the maximum diameter and DOI [2, 5, 8, 10, 21, 23, 24]; however, there is no investigation whether the radiological maximum diameter and DOI include peritumoral enhancement. The maximum diameter and DOI may be overestimated when peritumoral enhancement is included; therefore, the radiological maximum diameter and DOI should exclude peritumoral enhancement.

The T category based on the coronal CET1WI-MEP was a more relevant prognostic factor for recurrence than the T category based on pathological assessment. Pathological assessment incurs several problems. First, the oral tongue has a curved natural surface, and drawing an arbitrary straight line from the basement membrane of adjacent normal squamous epithelium may significantly underestimate the actual DOI [20]. Second, the pathological maximum diameter and DOI are measured from each section; however, in some cases, adjacent normal squamous mucosa is not present horizontally in the pathological specimen with the deepest level of invasion or in the entire specimen, or the adjacent uninvolved epithelium is present only on one side of the tumor section [7, 20]. Third, accurate assessment of the horizon is difficult in tumors where the natural contour of the epithelium is not uniform or straight [7]. Fourth, tongue tissue specimens are prone to distortion during handling [25]. Lastly, the section thickness of MRI is finer than the thickness of histological slides [25]. In contrast, the radiological maximum diameter and DOI can be measured by observing the whole tumor. Thus, radiological measurements may be more accurate than pathological measurements. This is the first study to determine the prognostic factor among pathological and radiological T staging using Kaplan–Meier survival analysis. However, further investigation is required.

Radiomics is a valuable tool for predicting prognosis for OTSCC; therefore, there is a paradigm shift to include radiomics to predict survival in OTSCC. A past study provides a new approach for OTSCC treatment management using a combined model based on clinical features and MRI radiomic parameters and concluded that the radiological T category was superior to the pathological T category in predicting prognosis [26]. Thus, if the true prognostic value of radiomics can be revealed, radiomics would become increasingly important in the future.

This study has several limitations. First, the cohort included a moderately small number of cases because the present study was conducted at a single institution; therefore, this study could not compare prognostic factors using stratifies cohort of patients with all T scores with and without metastases. Second, the maximum diameter and DOI on the combined axial/coronal T2WI or FST2WI could not be evaluated as coronal T2WI and axial FST2WI were not performed at our institution at the time of this study. Third, this study included patients with and without cervical nodal metastases. Fourth, disease-specific survival rates and overall survival were calculated in this study; however, we could not evaluate relative survival because of a lack of sufficient statistical knowledge. Finally, only two-dimensional MRI images were obtained in this study. If three-dimensional MRI images were used for evaluation, the maximum diameter and DOI could be measured more accurately.

In conclusion, the T category determined by MEP on coronal CET1WI was the best validated prognostic factor among all radiological and pathological T category measurements. Peritumoral enhancement on CET1WI should be excluded when measuring the radiological maximum diameter and DOI as the T category based on MEP was more strongly associated with patient prognosis than the T category based on MIP.
